# Fermented Aloreña Table Olives as a Source of Potential Probiotic *Lactobacillus pentosus* Strains

**DOI:** 10.3389/fmicb.2016.01583

**Published:** 2016-10-07

**Authors:** Beatriz Pérez Montoro, Nabil Benomar, Leyre Lavilla Lerma, Sonia Castillo Gutiérrez, Antonio Gálvez, Hikmate Abriouel

**Affiliations:** ^1^Área de Microbiología, Departamento de Ciencias de la Salud, Facultad de Ciencias Experimentales, Universidad de JaénJaén, Spain; ^2^Área de Estadística e Investigación Operativa, Departamento de Estadística e Investigación Operativa, Facultad de Ciencias Experimentales, Universidad de JaénJaén, Spain

**Keywords:** Aloreña table olives, *Lactobacillus pentosus*, probiotics, functional and technological properties, gut survival

## Abstract

A collection of 31 *Lactobacillus pentosus* strains isolated from naturally fermented Aloreña green table olives were screened in depth in the present study for their probiotic potential. Several strains could be considered promising probiotic candidates since they showed good growth capacity and survival under simulated gastro-intestinal conditions (acidic pH of 1.5, up to 4% of bile salts and 5 mM of nitrate), good ability to auto-aggregate which may facilitate their adhesion to host cells as multiple aggregates and the subsequent displacement of pathogens. Moreover, co-aggregation of lactobacilli with pathogenic bacteria was shown with *Listeria innocua, Staphylococcus aureus, Escherichia coli*, and *Salmonella* Enteritidis as good defense strategy against gut and food pathogens. Furthermore, they exhibited adherence to intestinal and vaginal cell lines, such property could be reinforced by their capacity of biofilm formation which is also important in food matrices such as the olive surface. Their antagonistic activity against pathogenic bacteria by means of acids and plantaricins, and also their different functional properties may determine their efficacy not only in the gastro-intestinal tract but also in food matrices. Besides their ability to ferment several prebiotics, the new evidence in the present study was their capacity to ferment lactose which reinforces their use in different food matrices including dairy as a dietary adjunct to improve lactose digestibility. *Lactobacillus pentosus* CF2-10N was selected to have the best probiotic profile being of great interest in further studies. In conclusion, spontaneous fermented Aloreña table olives are considered a natural source of potential probiotic *L. pentosus* to be included as adjunct functional cultures in different fermented foods.

## Introduction

Lactobacilli are Gram-positive, non-spore-forming rods or coccobacilli, catalase-negative (although some strains possess a pseudocatalase), aero-tolerant or anaerobic, aciduric, or acidophilic and nutritionally fastidious (Hammes and Vogel, 1995). *Lactobacillus* genus represents the largest and heterogeneous group among lactic acid bacteria “LAB.” Their large genome exhibit a high degree of physiology and genetic diversity which make them very attractive candidates to explore a wide variety of functional and technological properties with high impact in medical and industrial applications. In this sense, lactobacilli considered as generally recognized as safe (GRAS) in USA were largely used as starter and/or protective cultures in fermented vegetables, dairy products, sausages, and fish (Leroy and de Vuyst, 1999; Heller, 2001; Hansen, 2002; Holzapfel, 2002; Giraffa et al., 2010; Franz et al., 2011; Garrigues et al., 2013). This fact is due to their high acidification capacity and their ability to produce an arsenal of antimicrobial substances (organic acids, hydrogen peroxide, antifungal peptides and bacteriocins) (Ruiz-Barba et al., 1994; Holzapfel et al., 1995; Magnusson and Schnürer, 2001), and also to their crucial role in the rheology and texture properties of fermented food products via production of exopolysaccharides, aroma compounds and organic acids (O’Connor et al., 2005). Likewise, they were also used as probiotics since they are part of human microbiota (oral cavity, gastrointestinal tract, and vagina) exhibiting several beneficial effects on the host. However, some lactobacilli strains are known for their pathogenic potential (Cannon et al., 2005) and according to the Qualified Presumption of Safety (QPS) approach established by the European Food Safety Authority (EFSA, 2008), some *Lactobacillus* species have “QPS” status and could be used as probiotics such as *L. acidophilus, L. plantarum*, and *L. pentosus* among 35 species (EFSA, 2012, 2015), although a full *in vitro* safety assessment is required for each strain intended to be used in foods to ensure the absence of virulence determinants and transferable antibiotic resistance genes.

Probiotics include “good and live microorganisms” when administered in adequate amounts, benefit the host’s health (FAO/WHO, 2001). Among them, bacteria and specially LAB –mainly represented by *Bifidobacterium* and *Lactobacillus* genera– are the most used probiotics besides yeasts (Saulnier et al., 2009). Probiotics were highly consumed through history in many fermented foods such as dairy and vegetable-based foods (pickles, fermented table olives, sauerkraut, and kimchi) and now they represent a healthful ingredient for an increasingly health-conscious consumer. It’s usually known that isolation and selection of potential probiotic bacteria has been achieved for long time from feces and breast milk, in the last years several researches were focused on the search for new probiotic bacteria sources (Ranjan Swain et al., 2014; Saxami et al., 2016; Sornplang and Piyadeatsoontorn, 2016). In fact, vegetable products as new carrier matrices of probiotics are actually of increasing interest due to the increased demand for non-dairy probiotic products by lactose intolerant individuals, vegetarians, allergic, and dyslipidemic individuals (Granato et al., 2010; Ranadheera et al., 2010). Furthermore, probiotics of vegetable origin exhibit special survival characteristics due to the naturally presence of high amounts of prebiotics in plant material (oligosaccharides) which improve their functional efficacy with the increased resistance to acidic environment during gastric transit (Ranadheera et al., 2010). Probiotics from different vegetables foods such cabbage and table olives among others (Yoon et al., 2006; Abriouel et al., 2012; Patel et al., 2012, 2014; Peres et al., 2012) have promising future. In this sense, LAB isolated from naturally fermented Aloreña green table olives (Abriouel et al., 2012) that were mainly represented by *L. pentosus* were screened in depth in the present study for their probiotic potential. Our goal was to select the most robust strains as promising probiotics in intestinal and vaginal infections with the aim to carry out in the future genomic characterization of their probiotic potential.

## Materials and Methods

### Bacterial Strains and Growth Conditions

Thirty-one strains of *Lactobacillus pentosus* isolated by Abriouel et al. (2012) from Aloreña green table olives naturally fermented by four small–medium enterprises (SMEs) from Malaga (Spain) were used in this study (Supplementary Table S1). Selection of lactobacilli was done on the basis of the preliminary functional screening done by Abriouel et al. (2012). These strains were routinely cultured at 30°C in de Man Rogosa and Sharpe (MRS) broth (Fluka, Madrid, Spain) or agar under aerobic conditions for 24–48 h. Strains were kept in 20% glycerol at -80°C for long term storage.

### Safety Aspects

DNAse and gelatinase activities were determined as described by Lavilla-Lerma et al. (2013). Mucin degradation ability of lactobacilli was carried out as reported by Muñoz-Atienza et al. (2013). Production of biogenic amines from tyrosine, histidine, ornithine, or lysine (all of them from Sigma, Madrid) was done as described by Yousif et al. (2005) on MRS agar plates supplemented with the corresponding amino acids. With respect to hemolytic activity, overnight cultures of lactobacilli were streaked on the agar blood (Scharlab, Spain) and then incubated anaerobically at 37°C for 24 h.

### Survival in Different Conditions and Aggregation Properties

Survival under gastric conditions including low pH (1.5–3) and bile salt concentration (0–4% with increments of 1%) was done according to the methods described by Millette et al. (2008). For acid tolerance, 1 ml of overnight MRS broth cultures was inoculated onto 19 ml of simulated gastric fluid (3.2 g/l pepsin and 2 g/l NaCl) adjusted at different pH values (with 5M HCl) and then incubated for 30 min at 37°C. Viable counts (CFU/ml) were determined after incubation plating 1 ml of the mixture on MRS-agar. As reference, viable bacteria without simulated gastric was used (Millette et al., 2008). Regarding bile salt tolerance, MRS-agar plates added with different concentrations of bile salt mixture (Sigma B-3426) were inoculated onto the surface by overnight MRS broth cultures (100 μl). Then, the plates incubated at 37°C for 72 h were examined visually for bacterial growth (Millette et al., 2008).

Auto-aggregation capacity of lactobacilli was determined as reported by Vizoso Pinto et al. (2007). Overnight cultures (2 ml) of lactobacilli in MRS broth were harvested, washed and resuspended in sterile phosphate buffered saline (PBS). After 2 h at room temperature, 100 μl were removed from the top of the suspension and were transferred to a cuvette containing 900 μl PBS. The auto-aggregation percentage is expressed as: (1 -*A*_1_/*A*_0_) × 100, where *A*_0_ and *A*_1_ represent the absorbance measured at 580 nm at time = 0 and time = 2 h, respectively.

Co-aggregation capacity of lactobacilli with pathogenic bacteria (*Listeria innocua* CECT 910, *Staphylococcus aureus* CECT 4468, *Escherichia coli* CCUG 47553, and *Salmonella* Enteritidis UJ3449) was carried out according to Vlková et al. (2008). Overnight cultures (10 ml) of lactobacilli in MRS broth and pathogenic bacteria in TSB broth at 37°C were harvested, washed, resuspended in sterile PBS and their OD_600_ was adjusted to 1. Cell suspension was prepared mixing 3 ml of each bacteria (*L. pentosus* and one pathogenic strain) and then the OD_600_ of upper suspension was measured at time 0 and after 1 h incubation at room temperature. The percentage of co-aggregation was expressed as: Co-Agg% = [1 - (*A*_600_of upper suspension at time 1 h/*A*_600_of total bacterial suspension at time 0)] × 100.

Biofilm formation by lactobacilli was tested as described by Toledo-Arana et al. (2001). The OD_620_ was measured in microplate reader (Varioskan Flash Reader, Thermo Scientific) using 1% crystal violet.

### Technological Properties of Lactobacilli

The capacity of *L. pentosus* strains (1 × 10^6^ CFU/ml) to grow in MRS broth (Scharlab, Spain) under different conditions of temperature (4, 10, 30, and 37°C) and in the presence of salt (6.5% NaCl at 30°C) was tested and quantified determining viable cell number (CFU/ml) after 0, 1, 3, and 6 days of incubation. Survival capacity of lactobacilli to freezing temperature (at -80°C) was checked during 0, 1, 3, and 6 days of storage. In all cases, cell counts were done in triplicate on MRS agar (Scharlab, Spain) for 48 h at 30°C.

Screening of α-amylase, protease, bile salt hydrolase (BSH), haeme-dependent catalase and carboxymethyl cellulase (CMC) were tested as described by Knauf et al. (1992), Franz et al. (2001), Lucas et al. (2001), Ben Omar et al. (2004), and Yousif et al. (2005), respectively.

Regarding the utilization of non-digestible compounds, the α-galactoside sugars tested were stachyose or raffinose as described by Yousif et al. (2005). The plates were incubated at 30°C and observed for acid production every day over a 3-day period. With respect to oxalate degradation, lactobacilli were screened as reported by Gomathi et al. (2014) using the agar well-diffusion method. For this, 20 mM calcium oxalate plates were inoculated by 0.1 ml of lactobacilli overnight cultures and then incubated at 37°C for 12 h for clear zone observation around the wells.

On the other hand, growth of lactobacilli on prebiotics was done as described by Makras et al. (2005) using the agar plate assays. In this sense, modified MRS broth without glucose and supplemented with 0.5 g/l of L-cysteine hydrochloride (Sigma) (mMRS) was added with 2% (w/v) of different energy sources (glucose, fructose, galactose, lactose, saccharose, lactulose, or inulin) and 300 mg/l of bromocresol purple (Sigma) as a color indicator. Lactobacilli suspensions were prepared as described by Makras et al. (2005) and spotted on mMRS agar plates which were incubated anaerobically at 37°C for 48 h. Plates performed in triplicate were checked for color changes around the colonies.

### Antimicrobial Activity

Production of hydrogen peroxide was performed according to the method of Marshall (1979). Bacteriocin screening was done by the spot-on-a-lawn method as described by Abriouel et al. (2012). PCR screening of plantaricin genes was carried out as described by Ben Omar et al. (2006).

### Tolerance to Simulated Human GI Tract

Tolerance of selected *L. pentosus* strains -on the basis of their probiotic profile obtained by means of statistical methods (Principal Component Analysis) explained below- to simulated human gastrointestinal tract was carried out as reported by Chen et al. (2014) under simulated gastric juice (pH 3.0) and intestinal gastric juice (pH 8.0). Furthermore, we studied the effect of nitrate (5 mM) or glucose (500 mM) in both simulated gastrointestinal conditions.

### Adhesion to Cellular Lines

Selected *L. pentosus* strains with the best probiotic profile (simulated gastro-intestinal juice in standard conditions and in the presence of nitrate or glucose) were tested for their capacity to adhere to Enterocyte-like Caco-2 ECACC 86010202 (from colon adenocarcinoma) and HeLa 229 ECACC 86090201 (from vaginal cervix carcinoma) (both from the Scientific Instrument Services of the University of Granada, Spain). Eukaryotic cells were cultured as described by Lavilla-Lerma et al. (2013). Adhesion assays were carried out following the method of Moroni et al. (2006) by adding 250 μl (10^8^ CFU/mL) of each bacterial strain to a monolayer of differentiated cells (Lavilla-Lerma et al., 2013). Plates were then incubated at 37°C for 30 min and free bacteria were eliminated by washing the cell layers twice with phosphate-buffered saline (PBS, Sigma). To determine the CFU/ml of lactobacilli adhered to cells, those were harvested with EDTA-trypsin, centrifuged, and serially diluted in PBS before plating on agar-MRS.

### Statistical Analysis

All analyses were done in triplicate. Statistical analysis of data was accomplished using Excel 2007 program to determine the average data ± standard deviations. Statistical treatment of adhesion data was conducted by analysis of variances (ANOVA) in Statgraphics Centurion XVI, software using Shapiro–Wilk test and the Levene test to check data normality and the 2-sided Tukey’s test to determine the significance of differences between strains, where a *P*-value of <0.05 was considered statistically significant.

Principal Component Analysis (PCA) was used to emphasize variation and bring out strong differences in co-aggregation capacity of *L. pentosus* strains with Gram-negative and Gram-positive pathogens. On the other hand, we also used PCA analysis for selection of the best probiotic *L. pentosus* strains by using the following discriminating variables: survival at low pH of 1.5, auto-aggregation and co-aggregation with different pathogens.

## Results

### Evaluation of the Safety Aspects of *Lactobacillus pentosus* Strains

None of the strains analyzed in the present study showed positive results for safety aspects tested.

### Survival of *Lactobacillus pentosus* Strains under Different Gastric Conditions

Under gastric conditions, different viability rates were shown depending on the *L. pentosus* strain (**Table 1**). All *L. pentosus* strains were able to survive (>85–100%) at low pH (2–3), however at pH 1.5 only 8 of 31 strains showed high and statistically significant survival rates (86–97%). All *L. pentosus* strains were able to survive in the presence of 4% bile salt (**Table 1**).

**Table 1:** Survivability of *Lactobacillus pentosus* strains under gastric conditions.

Strains	Survival at different pH (%±SD^∗^)				Survival at different concentrations of bile salt (%)			
	1,5	2	2,5	3	1	2	3	4
*L. pentosus* AP2-11	76,33 ± 0,07^lm^	97,80 ± 0,34^fg^	100 ± 0,42^klmno^	100 ± 1,75^bcd^	+	+	+	+
*L. pentosus* AP2-15N	96,51 ± 0,17^t^	100 ± 0,26^jkl^	100 ± 0,37^klmn^	100 ± 0,67^fghijkl^	+	+	+	+
*L. pentosus* AP2-16N	75,55 ± 0,42^l^	95,38 ± 0,14^d^	100 ± 0,42^ijk^	100 ± 0,28^cdefghij^	+	+	+	+
*L. pentosus* AP2-17	71,46 ± 0,44^j^	100 ± 0,25^klmno^	100 ± 0,05^jklm^	100 ± 0,73^bcde^	+	+	+	+
*L. pentosus* AP2-18	60,20 ± 0,47^f^	100 ± 0,54^opq^	100 ± 0,38^mnop^	100 ± 0,09^bcde^	+	+	+	+
*L. pentosus* CF1-6	81,23 ± 0,63^n^	97,56 ± 0,22^f^	97,93 ± 0,51^d^	99,49 ± 0,50^bcdefgh^	+	+	+	+
*L. pentosus* CF1-20N	38,54 ± 0,26^c^	91,58 ± 0,58^b^	92,39 ± 0,37^a^	99,67 ± 0,35^bcdefghi^	+	+	+	+
*L. pentosus* CF1-23N	40,90 ± 0,44^d^	99,30 ± 0,39^hij^	100 ± 0,61^jkl^	100 ± 0,82^bc^	+	+	+	+
*L. pentosus* CF1-30	33,66 ± 0,33^a^	85,67 ± 0,12^a^	99,01 ± 0,91^defg^	98,16 ± 0,28^b^	+	+	+	+
*L. pentosus* CF1-33N	65,30 ± 0,82^i^	92,76 ± 0,13^c^	99,21 ± 1,01^efgh^	100 ± 0,54^s^	+	+	+	+
*L. pentosus* CF1-37N	83,58 ± 1,10^o^	100 ± 0,20^jklm^	100 ± 0,30^ghij^	100 ± 0,11^bcdefg^	+	+	+	+
*L. pentosus* CF1-38	65,83 ± 0,26^i^	100 ± 0,23^lmnop^	100 ± 0,17^ijk^	100 ± 0,19^pq^	+	+	+	+
*L. pentosus* CF1-39	61,44 ± 0,67^gh^	100 ± 0,48^pq^	100 ± 0,99^nop^	100 ± 0,02^mnop^	+	+	+	+
*L. pentosus* CF1-43N	60,97 ± 0,82^fg^	98,51 ± 0,11^fgh^	98,84 ± 1,03^def^	100 ± 0,23^hijklmn^	+	+	+	+
*L. pentosus* CF2-5	90,48 ± 0,31^q^	100 ± 0,38^s^	100 ± 0,09^q^	100 ± 0,00^efghijk^	+	+	+	+
*L. pentosus* CF2-9	77,21 ± 0,41^m^	99,64 ± 0,18^ijk^	99,81 ± 0,62^fghi^	98,71 ± 0,95^bcde^	+	+	+	+
*L. pentosus* CF2-10N	87,01 ± 0,62^p^	99,61 ± 0,50^ijk^	99,81 ± 0,32^fghi^	98,90 ± 0,38^bcdef^	+	+	+	+
*L. pentosus* CF2-11	90,70 ± 0,74^q^	98,74 ± 0,74^ghi^	100 ± 0,94^ijk^	100 ± 1,59^defghijk^	+	+	+	+
*L. pentosus* CF2-12	86,29 ± 0,25^p^	94,66 ± 0,69^d^	96,41 ± 0,27^c^	100 ± 0,43^qr^	+	+	+	+
*L. pentosus* CF2-15G	91,87 ± 0,36^r^	100 ± 0,50^pq^	100 ± 0,16^klmnop^	100 ± 1,71^mnop^	+	+	+	+
*L. pentosus* CF2-15P	87,23 ± 0,82^p^	96,54 ± 0,10^e^	94,79 ± 0,26^b^	100 ± 0,18^nop^	+	+	+	+
*L. pentosus* CF2-20G	40,86 ± 0,42^d^	100 ± 0,15^klmn^	100 ± 0,15^ijk^	100 ± 0,31^klmnop^	+	+	+	+
*L. pentosus* CF2-20P	73,85 ± 0,55^k^	100 ± 0,30^lmnop^	100 ± 0,43^ijk^	100 ± 0,64^bcdefgh^	+	+	+	+
*L. pentosus* LP1N	33,51 ± 0,77^a^	100 ± 0,75^r^	100 ± 1,16^p^	100 ± 0,42^ijklmno^	+	+	+	+
*L. pentosus* LP5N	71,26 ± 0,07^j^	97,57 ± 0,68^f^	100 ± 0,09^hij^	100 ± 1,63^ghijklm^	+	+	+	+
*L. pentosus* LP7N	61,78 ± 0,52^gh^	100 ± 0,52^qr^	100 ± 0,73^nop^	100 ± 0,53^rs^	+	+	+	+
*L. pentosus* LP8N	35,78 ± 0,25^b^	94,50 ± 0,29^d^	100 ± 0,49^op^	100 ± 0,52^klmnop^	+	+	+	+
*L. pentosus* MP-10	94,36 ± 0,20^s^	100 ± 0,06^mnop^	100 ± 0,65^lmnop^	100 ± 0,91^opq^	+	+	+	+
*L. pentosus* 2C5	62,24 ± 0,44^h^	100 ± 0,84^nopq^	100 ± 0,48^klmnop^	100 ± 1,45^jklmnop^	+	+	+	+
*L. pentosus* 5C2	37,83 ± 0,11^c^	98,86 ± 1,01^hi^	99,39 ± 0,26^efgh^	99,70 ± 0,09^a^	+	+	+	+
*L. pentosus* 5C3	42,67 ± 0,20^e^	94,71 ± 0,74^d^	98,24 ± 0,44^de^	100 ± 0,10^lmnop^	+	+	+	+

*±SD, standard deviations of three independent experiments.*

*^∗^Different lowercase letters represent significant differences according to 2-sided Tukey’s HSD between strains (*p* < 0.05)*

Auto-aggregation of lactobacilli belonging to the same strain is an important feature especially in the human gut. **Table 2** showed that 6, 13, and 12 of *L. pentosus* strains exhibited different auto-aggregation abilities ranging from high (50–77.92%), medium (35–50%), and low (16–35%), respectively (**Table 2**), taking as control *L. johnsonii* CECT 289 (35%). Variability in auto-aggregation ability was obtained among the tested strains (*p* < 0.05) belonging to the three groups mentioned above, indicating that auto-aggregation is a strain specific property.

**Table 2:** Auto-aggregation, co-aggregation, and biofilm formation abilities of *Lactobacillus pentosus* strains.

Strains	Auto-aggregation (% ± SD^∗^)	Co-aggregation (%±SD^∗^)				
		*Listeria innocua* CECT 910	*Staphylococcus aureus* CECT 4468	*Escherichia coli* CCUG 47553	*Salmonella* Enteritidis UJ3449	Biofilm formation capacity^∗∗^
*L. pentosus* AP2-11	56,68 ± 5,04^hijk^	13,65 ± 0,55^abc^	9,96 ± 0,69^ab^	28,59 ± 0,51^jkl^	14,24 ± 1,35^a^	+++
*L. pentosus* AP2-15N	66,21 ± 3,11^kl^	30,74 ± 3,32^hij^	18,58 ± 0,62^e^	14,82 ± 0,61^c^	14.65 ± 0,85^ab^	++
*L. pentosus* AP2-16N	77,92 ± 7,22^l^	32,49 ± 1,36^hijk^	15,29 ± 1,93^d^	14,31 ± 1,62^bc^	19,94 ± 1,39^de^	+++
*L. pentosus* AP2-17	25,20 ± 1,25^abc^	15,96 ± 2,47^bcde^	13,87 ± 2,17^cd^	32,11 ± 2,32^mn^	25,32 ± 1,03^gh^	+
*L. pentosus* AP2-18	36,31 ± 7,03^bcdefg^	34,34 ± 1,51^ijk^	45,16 ± 1,24^p^	36,22 ± 2,66^o^	40,28 ± 1,93^mno^	-
*L. pentosus* CF1-6	41,03 ± 8,86^cdefghi^	8,41 ± 1,05^a^	13,76 ± 1,44^cd^	41,33 ± 1,75^p^	21,72 ± 2,01^ef^	+++
*L. pentosus* CF1-20N	48,23 ± 6,29^fghij^	35,76 ± 3,62^jk^	41,29 ± 0,31^no^	43,55 ± 2,64^p^	39,31 ± 1,83^lmno^	+
*L. pentosus* CF1-23N	36,95 ± 3,83^cdefg^	33,45 ± 2,72^ijk^	29,86 ± 3,12^ij^	23,22 ± 2,57^fgh^	40,48 ± 1,93^no^	+
*L. pentosus* CF1-30	35,83 ± 3,88^bcdefg^	33,04 ± 3,03^ijk^	41,76 ± 1,36^o^	23,77 ± 1,75^ghi^	37,25 ± 3,74^lm^	+
*L. pentosus* CF1-33N	42,11 ± 6,03^cdefghi^	26,33 ± 4,30^gh^	46,83 ± 3,06^p^	52,62 ± 3,13^qr^	37,01 ± 3,42^l^	+++
*L. pentosus* CF1-37N	19,28 ± 1,42^ab^	29,07 ± 1,28^hi^	33,81 ± 0,18^kl^	49,87 ± 2,01^q^	19,43 ± 3,46^de^	+++
*L. pentosus* CF1-38	26,27 ± 2,51^abc^	21,71 ± 0,34^efg^	34,32 ± 1,62^kl^	31,32 ± 2,46^lmn^	29,02 ± 2,12^ij^	+++
*L. pentosus* CF1-39	16,03 ± 1,81^a^	22,02 ± 1,73^efg^	21,04 ± 2,53^ef^	21,82 ± 1,79^fg^	17,17 ± 1,66^abcd^	+
*L. pentosus* CF1-43N	24,81 ± 7,83^abc^	52,66 ± 1,54^m^	31,54 ± 0,82^jk^	49,67 ± 2,18^q^	47,24 ± 1,60^p^	+++
*L. pentosus* CF2-5	30,08 ± 4,57^abcde^	46,18 ± 0,72^l^	67,37 ± 0,23^s^	56,34 ± 0,99^s^	46,37 ± 1,65^p^	+
*L. pentosus* CF2-9	41,26 ± 4,93^cdefghi^	44,49 ± 1,82^l^	53,60 ± 3,39^q^	54,96 ± 2,18^rs^	37,06 ± 1,86^l^	+++
*L. pentosus* CF2-10N	39,50 ± 6,45^cdefgh^	46,27 ± 1,77^l^	58,02 ± 1,65^r^	51,37 ± 1,94^q^	41,10 ± 0,12^o^	+
*L. pentosus* CF2-11	60,73 ± 5,50^jkl^	43,99 ± 0,18^l^	51,60 ± 1,90^q^	33,05 ± 1,68^no^	45,22 ± 2,77^p^	-
*L. pentosus* CF2-12	52,42 ± 9,22^ghijk^	19,86 ± 1,70^cdef^	40,69 ± 2,46^no^	18,13 ± 2,29^de^	14,19 ± 2,38^a^	+
*L. pentosus* CF2-15G	57,21 ± 3,49^ijk^	12,78 ± 1,49^ab^	26,22 ± 1,96^gh^	41,92 ± 2,07^p^	22,48 ± 0,81^efg^	-
*L. pentosus* CF2-15P	31,69 ± 9,03^abcdef^	16,06 ± 2,65^bcde^	23,54 ± 1,57^fg^	11,37 ± 1,77^ab^	22,05 ± 1,30^ef^	-
*L. pentosus* CF2-20G	47,96 ± 3,32^fghij^	17,19 ± 1,07^bcdef^	35,43 ± 0,83^lm^	10,68 ± 0,46^a^	23,42 ± 3,03^fgh^	-
*L. pentosus* CF2-20P	43,95 ± 4,89^defghij^	23,73 ± 1,93^fg^	35,45 ± 2,13^lm^	29,51 ± 3,31^klm^	37,55 ± 0,43^lmn^	-
*L. pentosus* LP1N	46,28 ± 1,51^efghij^	13,16 ± 0,88^ab^	19,14 ± 3,34^e^	29,44 ± 0,92^klm^	18,55 ± 1,21^cd^	+
*L. pentosus* LP5N	29,88 ± 3,51^abcde^	18,07 ± 1,44^bcdef^	27,23 ± 1,87^hi^	16,52 ± 2,38^cd^	25,89 ± 1,94^hi^	-
*L. pentosus* LP7N	29,65 ± 5,44^abcde^	37,37 ± 2,01^k^	38,35 ± 2,48^mn^	26,78 ± 2,50^ijk^	31,79 ± 0,79^jk^	-
*L. pentosus* LP8N	32,99 ± 1,83^abcdef^	12,24 ± 0,74^ab^	29,08 ± 2,46^hij^	14,45 ± 1,03^bc^	16,22 ± 1,06^abc^	-
*L. pentosus* MP-10	16,66 ± 2,81^a^	20,16 ± 1,45^def^	13,45 ± 1,57^cd^	22,88 ± 0,86^fgh^	18,45 ± 1,12^cd^	-
*L. pentosus* 2C5	44,27 ± 6,47^defghij^	12,12 ± 1,89^ab^	12,51 ± 0,66^bcd^	20,02 ± 2,86^ef^	17,60 ± 2,24^bcd^	+++
*L. pentosus* 5C2	47,46 ± 8,38^fghij^	20,81 ± 0,77^efg^	9,18 ± 1,66^a^	27,81 ± 2,78^jk^	32,25 ± 3,10^k^	+
*L. pentosus* 5C3	27,76 ± 1,53^abcd^	14,41 ± 1,59^abcd^	12,25 ± 1,19^abc^	25,97 ± 1,65^hij^	30,45 ± 0,38^jk^	-

*±SD, standard deviations of three independent experiments.*

*^∗^Different lowercase letters represent significant differences according to 2-sided Tukey’s HSD between strains (*p* < 0.05).*

*^∗∗^The corresponding categories of biofilm formation capacity measured by optical density at 595 nm: (–), non-biofilm forming (OD_*595*_ ≤ 1); (+), weak biofilm forming (1 < OD_*595*_ ≤ 2); (++), medium biofilm forming (2 < OD_*595*_ ≤ 3); (+++), strong biofilm forming (OD_*595*_ > 3) according to Toledo-Arana et al. (2001).*

**FIGURE 1:**
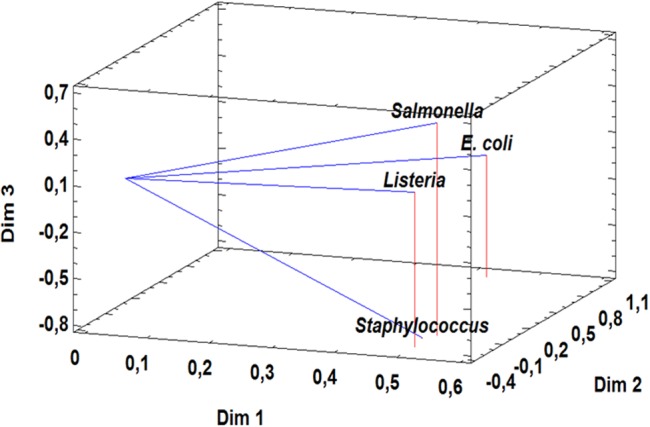
**Principal Component Analysis of co-aggregation capacity with Gram-positive and Gram-negative pathogens (*Listeria innocua* CECT 910, *Staphylococcus aureus* CECT 4468, *Escherichia coli* CCUG 47553, *Salmonella* Enteritidis UJ3449) for 31 *L. pentosus* strains**.

Co-aggregation of lactobacilli with pathogenic bacteria was variable and statistically significant depending on the lactobacilli and pathogenic strains used (**Table 2**). High co-aggregation capacity (40–67%) of lactobacilli (nine strains) was detected with *E. coli* and *S. aureus*, while five and six lactobacilli strains highly co-aggregated with *Listeria innocua* and *Salmonella*, respectively (**Table 2**). However, the other lactobacilli strains (39–48%) showed variable co-aggregation capacities ranging from 20 to 38% with all pathogenic bacteria. Furthermore, 23–39% of lactobacilli showed less than 20% of co-aggregation capacity (**Table 2**). In general, *L. pentosus* strains showed higher and statistically significant co-aggregation capacity with Gram-negative bacteria as compared to Gram-positive bacteria as shown by a multivariate analysis (PCA) with three components explaining 93.88% of total variation (**Figure 1**).

Regarding biofilm formation, 20 of 31 of *L. pentosus* strains were able to form biofilms although with different degree being 9 of 20 strains with high capacity (**Table 3**).

**Table 3:** Phenotypic and genotypic bacteriocinogenic activity of *Lactobacillus pentosus* strains isolated from fermented Aloreña Table olives.

Strains	Phenotypic activity of *Lactobacillus pentosus* strains against indicator bacteria^∗^						Genotypic detection of bacteriocin genes								
	*Listeria innocua* CECT 910	*Staphylococcus aureus* CECT 4468	*Enterococcus faecalis* S-47	*Bacillus cereus* LWL1	*Escherichia coli* CCUG 47553	*Salmonella* Enteritidis UJ3449	*plnA*	*plnB*	*plnC*	*plnD*	*plnEF*	*plnG*	*plnI*	*plnJ*	*plnK*	*plnN*	*plnNC8*	*plnS*	*plnW*
*L. pentosus* AP2-11	++	+	+	+	+	+	-	-	-	-	-	-	-	-	-	-	-	-	-
*L. pentosus* AP2-15N	++	+	+	+	+	+	-	-	-	-	-	-	-	-	-	-	-	-	-
*L. pentosus* AP2-16N	++	++	+	++	+	+	-	-	-	-	-	-	-	-	-	-	-	-	-
*L. pentosus* AP2-17	++	+	+	++	++	++	+	-	-	-	-	-	-	-	-	-	-	-	-
*L. pentosus* AP2-18	++	+	+	+	++	++	+	-	-	-	-	-	-	-	-	-	-	-	-
*L. pentosus* CF1-6	+	+	+	-	+	+	-	-	-	-	-	-	-	-	-	-	-	-	-
*L. pentosus* CF1-20N	++	++	++	+	+	++	+	-	-	-	-	-	-	-	-	-	+	-	+
*L. pentosus* CF1-23N	++	+	++	+	+	+	+	-	-	-	-	-	-	+	-	-	-	-	-
*L. pentosus* CF1-30	++	+	+	+	+	+	+	-	-	-	-	-	-	-	-	-	-	-	-
*L. pentosus* CF1-33N	++	++	+	+	+	+	+	-	-	-	-	-	-	-	-	-	-	-	-
*L. pentosus* CF1-37N	++	+	+	+	+	+	+	-	-	-	-	-	-	-	-	-	-	-	-
*L. pentosus* CF1-38	++	+	+	+	-	+	+	-	-	+	-	-	-	-	-	-	-	-	-
*L. pentosus* CF1-39	++	++	+	+	+	+	+	-	-	-	-	-	-	-	-	-	-	-	-
*L. pentosus* CF1-43N	++	+	+	+	+	+	+	-	-	-	-	-	-	-	-	-	-	-	-
*L. pentosus* CF2-5	++	++	++	+	+	+	+	-	-	+	-	-	-	-	-	-	-	-	-
*L. pentosus* CF2-9	++	+	+	+	+	+	+	-	-	+	-	-	-	-	-	-	-	-	-
*L. pentosus* CF2-10N	++	+	+	+	+	+	+	-	-	+	-	-	-	-	-	-	-	-	-
*L. pentosus* CF2- 11	++	+	+	-	-	+	+	-	-	+	-	-	-	-	-	-	-	-	-
*L. pentosus* CF2-12	++	++	-	+	+	+	-	-	-	-	-	-	-	+	-	-	+	-	+
*L. pentosus* CF2-15G	++	++	+	+	+	++	-	-	-	+	-	-	-	-	-	-	-	-	-
*L. pentosus* CF2-15P	++	++	+	+	+	+	-	-	-	+	-	-	-	-	-	-	-	-	-
*L. pentosus* CF2-20G	++	+	+	+	++	+	-	-	-	-	-	-	-	-	-	-	+	-	-
*L. pentosus* CF2-20P	++	++	+	++	+	+	-	-	-	-	-	-	-	-	-	-	-	-	-
*L. pentosus* Lp-1N	+	+	+	+	+	+	-	-	-	-	-	-	-	-	-	-	-	-	-
*L. pentosus* Lp-5N	++	++	+	+	+	-	-	-	-	-	-	-	-	-	-	-	-	-	-
*L. pentosus* Lp-7N	++	++	+	-	+	+	-	-	-	-	-	-	-	-	-	-	-	-	-
*L. pentosus* Lp-8N	++	++	+	++	+	+	-	-	-	-	-	-	-	-	-	-	-	-	-
*L. pentosus* MP-10	++	++	++	+	+	++	-	-	-	-	-	-	-	-	-	-	-	-	-
*L. pentosus* 2C5	++	+	+	+	+	+	-	-	-	-	-	-	-	-	-	-	-	-	-
*L. pentosus* 5C2	++	++	++	+	+	++	-	-	-	-	-	-	-	-	-	-	-	-	-
*L. pentosus* 5C3	+	+	+	+	-	-	-	-	-	-	-	-	-	-	-	-	-	-	-

*^∗^Inhibitory activity against indicator bacteria measured from the edge of the spot are indicated as + (inhibition zone of 1–3 mm) or as ++ (inhibition zone >3 mm).*

### Functional Properties of Lactobacilli

All *L. pentosus* strains were able to grow in the presence of 6.5% NaCl (data not shown). Supplementary Table S1 showed that all lactobacilli generally showed good survival capacity under different temperature conditions being growth capacity mainly dependent on the incubation temperature and the *L. pentosus* strain. Generally, under temperatures of 4, 10, 30, and 37°C, all strains showed growth after 1–6 days incubation by almost 2.74 Log_10_ units reaching the maximum after 1 day incubation at 30 or 37°C (except few cases) and 3 days at 10°C (Supplementary Table S1). However, at freezing temperature (-80°C) no growth was recorded and survival of almost all *L. pentosus* strains during storage for 6 days was shown (Supplementary Table S1). In this sense, almost all *L. pentosus* strains showed high survival capacity of 100%, however six strains showed a slight decrease in viable cell counts by 1.04–1.65 Log_10_ units after 6 days storage at -80°C (Supplementary Table S1).

The results obtained showed that all lactobacilli strains were able to produce BSH, 58 and 39% of strains were able to produce haeme-dependent catalase and cellulolytic activity, respectively (Supplementary Table S2). However, none of lactobacilli strains produced α-amylase nor protease (data not shown). Regarding fermentation of human non-digestible α-galactoside sugars, 52% of *L. pentosus* strains exhibited the capacity to ferment raffinose, but not stachyose except *L. pentosus* MP-10 (Supplementary Table S2). Furthermore, no oxalate degradation ability was found in *L. pentosus* strains (data not shown). Concerning growth of lactobacilli on prebiotics, all strains fermented the monosaccharides glucose, fructose, and galactose (except *L. pentosus* CF2-12 for galactose) (Supplementary Table S2). Moreover, all lactobacilli ferment saccharose and lactulose and almost all lactobacilli ferment lactose except three *L. pentosus* strains (CF2-12, Lp-7N, and 5C3) but none of the strains ferment inulin (Supplementary Table S2).

Regarding antimicrobial activity, none of lactobacilli strains produced hydrogen peroxide (data not shown), however bacteriocin activity was detected in all strains by means of phenotypic methods (**Table 3**). However, genotypic screening of plantaricin genes indicated the presence of *plnA* and *plnD* genes in 45 and 23% of *L. pentosus* strains, respectively (**Table 3**). Concerning other plantaricin genes, 6–10% of strains showed the presence of *plnJ, planNC8*, or *plnW* genes. Nevertheless, none of the strains exhibited the presence of *plnB, plnC, plnEF, plnG, plnI, plnK, plnN*, or *plnS* genes (**Table 3**).

### Tolerance to Simulated Human GI Tract

**FIGURE 2:**
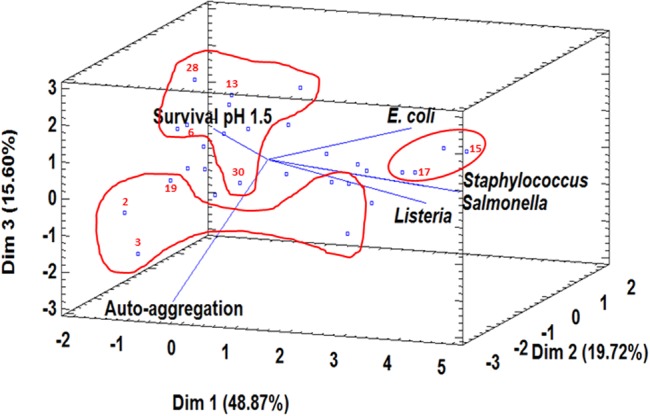
**Principal Component Analysis of six probiotic parameters (survival at pH 1.5, auto-aggregation, co-aggregation with *Listeria innocua* CECT 910, co-aggregation with *Staphylococcus aureus* CECT 4468, co-aggregation with *Escherichia coli* CCUG 47553, co-aggregation with *Salmonella* Enteritidis UJ3449) and the position of 31 *L. pentosus* strains**. Three groups with the best scores according to three components (Dim 1, Dim 2, and Dim 3) were surrounded in red and the most representative strains (2, *L. pentosus* AP2-15N; 3, *L. pentosus* AP2-16N; 6, *L. pentosus* CF1-6; 13, *L. pentosus* CF1-39; 15, *L. pentosus* CF2-5; 17, *L. pentosus* CF2-10N; 19, *L. pentosus* CF2-12; 28, *L. pentosus* MP-10; 30, *L. pentosus* 5C2) were selected to be used in further studies.

To carry out this test, 9 of 31 *L. pentosus* strains with the best probiotic profile were selected by using PCA analysis as described in “Materials and Methods” section. **Figure 2** represents the distribution of variables in a three dimensional analysis of the Principal Component (84.19% total variance) and also the position of *L. pentosus* strains in the space of three dimensions being organized in three main groups. Strains with the best scores were selected as the most representative strains (nine strains in total) to be used in further studies (**Figure 2**; **Table 4**). Under gastric conditions (pH 3.0), nine selected *L. pentosus* strains exhibited different survival rates depending on the strain and the exposure time (1–3 h) (**Table 4A**). After 3 h incubation in standard conditions (pH 3.0), *L. pentosus* AP2-15N, CF1-39, CF2-10N, CF2-12, and MP-10 strains showed high and statistically significant survival capacity of 96.96–99.76% (**Table 4A**). However, the rest of *L. pentosus* strains (AP2-16N, CF1-6, CF2-5, and 5C2) showed 58.62–81.85% survival under standard conditions (**Table 4A**). Similar results were obtained under simulated intestinal conditions (pH 8.0) for all strains (**Table 4B**).

**Table 4:** Survival of *Lactobacillus pentosus* strains under simulated gastric juice at pH 3 **(A)** and intestinal juice at pH 8 **(B)** and the effects of nitrate and glucose.

Strains	Standard			+ Nitrate 5 mM (p/v)			+ Glucose 500 mM (p/v)		
	1 h	2 h	3 h	1 h	2 h	3 h	1 h	2 h	3 h
**(A) Survival rate (% ± SD^∗^) in simulated gastric juice (pH 3)**						
*L. pentosus* AP2-15N	98,74 ± 0,12^c^	98,36 ± 0,30^e^	98,67 ± 0,21^f^	99,01 ± 0,19^cd^	98,63 ± 0,33^e^	98,61 ± 0,12^e^	99,28 ± 0,57^b^	99,48 ± 0,42^c^	99,67 ± 0,10^a^
*L. pentosus* AP2-16N	92,19 ± 0,08^a^	78,29 ± 0,58^b^	65,07 ± 0,97^b^	94,75 ± 0,35^a^	79,98 ± 0,41^c^	78,67 ± 0,42^c^	102,38 ± 0,41^d^	102,66 ± 0,05^e^	102,59 ± 0,68^cd^
*L. pentosus* CF1-6	93,11 ± 0,84^b^	84,52 ± 0,25^d^	81,85 ± 0,51^d^	96,01 ± 0,43^b^	84,58 ± 0,44^d^	81,86 ± 0,10^d^	99,00 ± 0,18^ab^	98,14 ± 0,11^b^	100,62 ± 0,10^ab^
*L. pentosus* CF1-39	100,86 ± 0,41^d^	100,04 ± 0,37^fg^	97,67 ± 0,66^ef^	101,38 ± 0,33^e^	101,18 ± 0,04^g^	99,13 ± 0,12^f^	103,00 ± 0,12^d^	103,17 ± 0,20^ef^	100,30 ± 1,53^a^
*L. pentosus* CF2-5	102,76 ± 0,95^e^	75,51 ± 0,99^a^	58,62 ± 0,58^a^	104,18 ± 0,15^f^	66,65 ± 0,06^a^	43,12 ± 0,26^a^	102,48 ± 0,11^d^	103,84 ± 0,32^f^	102,08 ± 1,23^bc^
*L. pentosus* CF2-10N	98,85 ± 0,49^c^	101,06 ± 0,30^g^	98,30 ± 0,35^f^	99,58 ± 0,48^d^	100,83 ± 0,31^g^	98,39 ± 0,09^e^	100,41 ± 0,27^c^	100,91 ± 0,36^d^	99,94 ± 0,09^a^
*L. pentosus* CF2-12	99,83 ± 0,28^cd^	99,91 ± 0,33^f^	99,76 ± 0,14^g^	98,26 ± 0,21^c^	98,21 ± 0,16^e^	98,25 ± 0,08^e^	99,25 ± 0,34^b^	99,28 ± 0,30^c^	99,12 ± 0,17^a^
*L. pentosus* MP-10	99,90 ± 0,71^cd^	99,83 ± 0,46^f^	96,96 ± 0,19^e^	101,11 ± 0,55^e^	100,19 ± 0,23^f^	100,88 ± 0,31^g^	98,42 ± 0,16^a^	99,12 ± 0,92^c^	99,60 ± 0,04^a^
*L. pentosus* 5C2	100,05 ± 0,85^cd^	83,19 ± 0,45^c^	77,07 ± 0,10^c^	98,39 ± 0,03^c^	73,54 ± 0,21^b^	55,69 ± 0,20^b^	104,89 ± 0,21^e^	94,48 ± 0,05^a^	104,07 ± 0,00^d^
**(B) Survival rate (% ± SD^∗^) in simulated intestinal juice (pH 8)**						
*L. pentosus* AP2-15N	97,69 ± 0,09^f^	96,47 ± 0,18^f^	95,78 ± 0,41^f^	98,23 ± 0,13^f^	85,83 ± 0,57^f^	85,18 ± 0,15^d^	99,50 ± 0,20^b^	99,72 ± 0,17^b^	99,86 ± 0,16^c^
*L. pentosus* AP2-16N	64,37 ± 0,22^a^	64,04 ± 0,22^a^	64,24 ± 0,75^a^	78,09 ± 0,22^d^	77,81 ± 0,44^e^	72,17 ± 0,13^c^	102,95 ± 0,21^d^	102,65 ± 0,64^e^	103,07 ± 0,25^e^
*L. pentosus* CF1-6	79,80 ± 0,13^d^	76,21 ± 0,16^d^	73,61 ± 0,64^c^	79,98 ± 0,37^e^	74,72 ± 0,66^d^	72,94 ± 0,14^c^	99,13 ± 0,49^b^	99,82 ± 0,23^b^	99,53 ± 0,09^bc^
*L. pentosus* CF1-39	100,59 ± 0,51^h^	100,64 ± 0,15^h^	99,57 ± 0,74^g^	100,54 ± 0,43^g^	100,51 ± 0,34^h^	99,26 ± 0,20^f^	101,17 ± 0,38^c^	100,95 ± 0,07^c^	101,95 ± 0,20^d^
*L. pentosus* CF2-5	66,07 ± 0,09^b^	68,95 ± 0,58^b^	70,26 ± 0,47^b^	43,58 ± 0,53^a^	43,62 ± 0,96^a^	47,04 ± 0,40^a^	104,81 ± 0,80^e^	104,63 ± 0,05^f^	114,25 ± 0,17^g^
*L. pentosus* CF2-10N	99,81 ± 0,26^gh^	99,27 ± 0,17^g^	99,80 ± 0,03^g^	100,05 ± 0,21^g^	100,00 ± 0,38^h^	100,21 ± 0,12^f^	102,24 ± 0,08^c^	101,88 ± 0,06^d^	102,06 ± 0,06^d^
*L. pentosus* CF2-12	99,61 ± 0,05^g^	99,57 ± 0,06^g^	99,65 ± 0,16^g^	98,17 ± 0,16^f^	94,92 ± 0,68^g^	94,57 ± 0,50^e^	98,94 ± 0,18^b^	99,25 ± 0,13^b^	99,13 ± 0,11^b^
*L. pentosus* MP-10	94,60 ± 0,60^e^	95,14 ± 1,07^e^	92,97 ± 1,06^e^	71,47 ± 0,09^c^	71,65 ± 0,99^c^	71,98 ± 0,97^c^	96,75 ± 0,46^a^	97,96 ± 0,42^a^	97,17 ± 0,22^a^
*L. pentosus* 5C2	70,71 ± 0,61^c^	73,46 ± 0,26^c^	76,19 ± 0,26^d^	52,99 ± 0,42^b^	48,53 ± 0,08^b^	50,45 ± 0,44^b^	104,16 ± 0,80^de^	108,42 ± 0,56^g^	112,49 ± 0,64^f^

*±SD, standard deviations of three independent experiments.*

*^∗^Different lowercase letters represent significant differences according to 2-sided Tukey’s HSD between strains (*p* < 0.05).*

When simulated gastric juice was supplemented with 5 mM nitrate, different results were obtained depending on the *L. pentosus* strain and simulated gastrointestinal conditions. Generally, reduction of survival capacity was observed in the same lactobacilli strains which showed poor survival capacity under gastric and intestinal conditions (**Table 4**).

To evaluate the effect of glucose on survival capacity of lactobacilli under gastric and intestinal conditions, 500 mM glucose was added to simulated gastric (pH 3.0) and intestinal (pH 8.0) juices (**Table 4**). The results obtained showed that glucose plays a protective role of lactobacilli under both conditions since all strains reached almost 100% (97–100%) survivability after 3 h incubation in simulated gastric juice (pH 3.0) and simulated intestinal juice (pH 8.0) (**Table 4**).

In conclusion, *L. pentosus* strains (CF2-5 and 5C2) showed less survival capacity in simulated gastro-intestinal juice in the presence of 5 mM nitrate, thus they were discarded from further analysis.

### Adhesion to Cellular Lines

**FIGURE 3:**
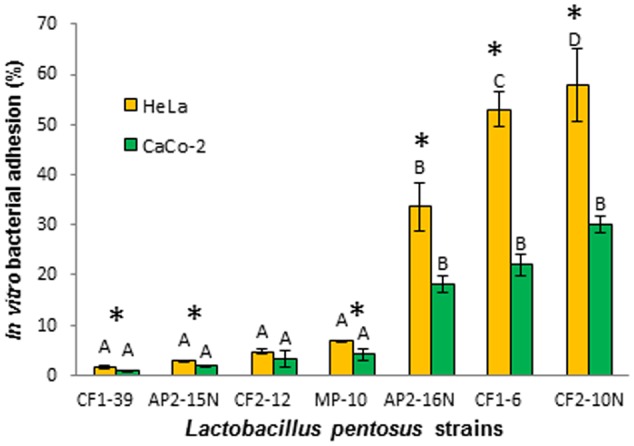
**Adhesion of *Lactobacillus pentosus* strains to Caco-2 (A) and HeLa 229 (B) cell lines.** Different letters represent significant differences according to 2-sided Tukey’s test (*p* < 0.05) measured between strains for each cellular line. Asterisks indicated significant differences according to 2-sided Tukey’s test (*p* < 0.05) measured for each strain in both cellular lines.

Selected *L. pentosus* strains with the best probiotic profile (seven strains) were tested for their capacity to adhere to Enterocyte-like Caco-2 ECACC 86010202 (from colon adenocarcinoma) and HeLa 229 ECACC 86090201 (from vaginal cervix carcinoma). The results obtained showed a high variability in adhesion capacity depending on the strain and also on the cellular line (**Figure 3**) since the adhesion to HeLa 229 (up to 57.88%) was more important and statistically significant than to Caco-2 (30.02%) cells as shown in **Figure 3** except for *L. pentosus* CF2-12. Thus, *L. pentosus* strains showed decreasing adhesion capacity to Hela cells as follows: CF2-10N > CF1-6 > AP2-16N > group of MP-10, CF2-12, AP2-15N and CF1-39 strains (**Figure 3**). However, in the case of Caco-2 cells, two groups were defined: one comprising *L. pentosus* AP2-16N, CF1-6, and CF2-10N strains and the other group with the rest of strains being statistically different (**Figure 3**). In conclusion, *L. pentosus* CF2-10N, CF1-6, and AP2-16N strains exhibited the best adhesion profile (33.55–57.88% and 18.11–30.02% for HeLa 229 and Caco-2 cells, respectively).

## Discussion

There is a growing interest in developing non-dairy probiotic products due to vegetarianism emergence, lactose intolerance, cholesterolemia, and allergy (Granato et al., 2010; Ranadheera et al., 2010). Recently, several researches were focused on selection of non-dairy probiotics especially from vegetables, fruits, and cereals (Peres et al., 2012; Martins et al., 2013). In this way, spontaneous fermented Aloreña table olives are considered a natural source of active and viable microorganisms (LAB and yeasts) (Abriouel et al., 2011) and a promising vehicle of potential probiotic LAB on the basis of the preliminary tests (Abriouel et al., 2012). Furthermore, some studies showed that *Lactobacillus* species adhere effectively to the surface of olives during storage as biofilms protecting the fruits from alteration and colonization by undesirable planktonic microorganisms such as fungi (Faten et al., 2016). Besides their nutritional value (unsaturated fatty acids, fiber, vitamins, minerals, flavonoids, and polyphenols), the presence of probiotic LAB able to survive during storage provides Aloreña table olives an added value. Moreover, fortification of previously fermented olives with the autochthonous putative probiotic lactobacilli may be a good strategy due to the adherence of lactobacilli to the surface of fruits which are the real food finally ingested by consumers (Rodríguez-Gómez et al., 2014).

In the present study, analysis in depth of probiotic features carried out on 31 *L. pentosus* strains isolated from the fermentation of Aloreña table olives (Abriouel et al., 2012) showed that some strains has a promising future to be used as probiotics in table olives or other food matrices. Survival and growth of *L. pentosus* strains under different temperature conditions (4, 10, 30, and 37°C) was monitored during several days (7–9 log_10_ CFU/ml) especially at low temperature of 4 and 10°C highlighting the possibility of maintaining high viable number of microorganisms throughout the entire shelf-life of the products. Good survival capacity was also observed in *L. pentosus* strains at freezing temperature of -80°C of 7–9 log_10_ CFU/ml. In general, lactobacilli isolated from Aloreña table olives maintained survival capacity at different temperature conditions which is in accordance with the number of viable cells shown to be efficacious in probiotic foods (6–8 log_10_ CFU/ml), although some probiotics from other food matrices showed poor survivability (Dunne et al., 2001; Gueimonde et al., 2004). However, under gastric conditions (pH and bile salt), survivability was highly dependent on the strain studied especially at low pH (1.5). Tolerance to acidity was shown in all *L. pentosus* strains (pH 2–3), although eight strains showed high survivability (86–97%) at pH 1.5. Furthermore, all *L. pentosus* strains were able to survive in the presence of 4% bile salt, such concentration is considered higher than the normal intestinal concentration (2%). Other features than the ability to survive in the presence of acids and bile salts are also important in probiotics such as the auto-aggregation and co-aggregation capacities and biofilm formation. In this way, auto-aggregation and co-aggregation of *L. pentosus* strains were shown to be strain-specific involving most probably strain-specific surface proteins such as mucus binding, aggregation promoting and intracellular adhesion. To clarify this fact, further studies should be carried out by means of genomic analysis in a similar way as was reported in *L. pentosus* KCA1 isolated from healthy woman vagina (Anukam et al., 2013). The 19% of *L. pentosus* strains exhibited high ability to auto-aggregate (50–77.92%) being 42% of the strains with medium auto-aggregation capacity (35–50%) which is important in their adhesion to host cells as multiple aggregates and the subsequent displacement of pathogens. Similarly, Botta et al. (2014) obtained 11.8 to 49.4% of auto-aggregation capacity in *L. pentosus* strains isolated from Sicilian table olives, however, in the present study some strains were able to auto-aggregate up to 77.92%. However, other lactobacilli isolated from Portuguese table olives (*L. plantarum* and *L. paraplantarum*) showed lower auto-aggregation capacities of 4–12% (Peres et al., 2014). Moreover, co-aggregation of lactobacilli with pathogenic bacteria is a good defense strategy against gut pathogens especially *E. coli, Salmonella, Listeria innocua*, and *S. aureus* tested in the present study and the results obtained were also strain dependent as was reported by Peres et al. (2014) for lactobacilli isolated from Portuguese table olives. Biofilm formation is also an important probiotic feature not only in epithelial cells but also on the olive surface for the reasons exposed above. In this study, several strains showed high capacity for biofilm formation.

Several studies showed that aggregation, adhesion and biofilm formation by lactobacilli was largely correlated with the presence of surface proteins (sortase-dependent proteins “SDPs,” mucus binding protein, aggregation promoting proteins, and intracellular adhesion proteins), polysaccharides and also their cell wall architecture (Granato et al., 1999; Kleerebezem et al., 2003). In this sense, several authors reported that SDPs was involved in auto-aggregation, biofilm formation and adhesion of lactobacilli to intestinal (van Pijkeren et al., 2006; Denou et al., 2008; Muñoz-Provencio et al., 2012) and vaginal epithelial cell lines (Malik et al., 2013). Besides the strain-specific properties, the physicochemical properties of the bacterial cell may be influenced by environmental conditions and thus influence the microbe–microbe or host–microbe interactions (Sengupta et al., 2013).

Regarding the functional properties of *L. pentosus* strains, several enzymes were produced such as BSH, haeme-dependent catalase, cellulase, α-galactosidase, and β-galactosidase. Furthermore, all lactobacilli were able to ferment several carbohydrates such as glucose, fructose, galactose, saccharose, and lactose (except two strains) and also they fermented the prebiotic lactulose (except one strain) but not inulin. Prebiotics as indigestible substances which stimulate healthy intestinal microbiota mainly lactobacilli and bifidobacteria includes several oligosaccharides, inulin, lactulose, lactosucrose, among others (Fric, 2007). In the present study, the presence of lactulose degrading enzyme and lactase in almost all lactobacilli is of great importance not only in the intestinal tract where they may ferment lactulose and grow but also they may improve lactose intolerance via fermentation in intolerant-lactose consumers, and thus those lactobacilli could be proposed as a dietary adjunct for milk to aid lactose digestion in humans as reported by Kim and Gilliland (1983) for *L. acidophilus*. Moreover, galacto-oligosaccharides (GOS) known as prebiotics maybe produced by the action of β-galactosidase on lactose via glycosyl transfer reactions which in turn is advantageous for their own proliferation and those of intestinal tract but this fact depends on the source of the β-galactosidase (Sako et al., 1999). In this sense, several reports described the production of β-galactosidase by *L. pentosus* strains isolated from different fermented foods (Pérez Pulido et al., 2007; Hemmaratchirakul et al., 2015), however, it is noteworthy to highlight that *L. pentosus* strains from table olives possess enzymes such as lactase that is not necessary in their own ecosystem since olives are free of lactose. The presence of genes coding for enzymes related with other ecosystems such as dairy products, may suggest the evolutionary relationship of lactobacilli colonizing different ecosystems. On the other hand, *L. pentosus* strains exhibited broad antimicrobial spectrum against Gram-positive and Gram-negative organisms including pathogens, being attributed to various extracellular metabolites such as lactic acid and bacteriocins as evidenced by the presence of several genes coding for plantaricins although the presence of plantaricin loci are not always related with bacteriocin production (Diep et al., 2009) and hence further studies are required to confirm plantaricin production. Bacteriocin production is a desirable trait in probiotic bacteria as defense mechanism in gastrointestinal tract against pathogens, but also in the added-probiotic food matrix to protect it from alteration and microbial colonization.

Selected *L. pentosus* strains on the basis of their probiotic profile (the most discriminative parameters since they showed similar results for example for bile salt tolerance, antimicrobial activity and some technological properties) showed high acid tolerance being able to survive in both simulated GI tract (pH 3.0 and pH 8.0) in the presence or absence of 5 mM nitrate, a concentration compatible with levels found in the upper intestinal tract of healthy volunteers and with values measured in the mouse intestinal mucus (Jones et al., 2007). However, such survivability was highly dependent on the strain tested. Matsumoto et al. (2004) reported that the acid tolerance of bacteria was related to the induced H^+^-ATPase activity. However, the effect of glucose addition improved the survivability of all *L. pentosus* strains including those that have exhibited reduction in viable rates. Acid tolerance of the lactobacilli is not only important in gastrointestinal conditions but also in acidic food matrices where lactobacilli may be added as adjuncts and the addition of glucose may be good strategy to ensure their survival. Furthermore, the seven *L. pentosus* strains (AP2-15N, AP2-16N, CF1-6, CF1-39, CF2-10N, CF2-12, and MP-10) selected showed different adhesion properties to Caco-2 and HeLa 229 cell lines being *L. pentosus* CF2-10N, CF1-6, and AP2-16N the most promising probiotics. *Lactobacillus pentosus* strains isolated from Aloreña table olives exhibited higher adherence to Caco-2 cells than *L. pentosus* strains isolated from fermented radish (19%) as reported by Damodharan et al. (2015) and also more than the reported probiotic and commercial *L. plantarum* WCFS1 strain (Jensen et al., 2012). Statistical analysis showed that *L. pentosus* strains exhibited significant differences in adherence to both cellular lines suggesting that *L. pentosus* CF2-10N, CF1-6, and AP2-16N shared the same mechanism of adherence being different to the other strains tested in the present study thus involving different adherence molecules.

## Conclusion

*Lactobacillus pentosus* strains isolated from naturally fermented Aloreña table olives could be considered promising probiotic candidates since they showed good growth capacity and survival under different environmental and gastro-intestinal conditions, good ability to auto-aggregate and co-aggregate with pathogenic bacteria, adherence to intestinal and vaginal cells, antagonistic activity and also they exhibited different functional properties determining their efficacy not only in the gastro-intestinal tract but also in food matrices. Besides their ability to ferment several prebiotics, the new evidence in the present study was their capacity to ferment lactose which reinforces their use in different food matrices containing lactose and thus to improve lactose digestibility, although further studies are required. *Lactobacillus pentosus* CF2-10N, CF1-6, and AP2-16N were selected as the most robust probiotic strains according to their high potential in several probiotic tests.

## Author Contributions

Conceived, designed the experiments, and drafted the paper: HA, NB, and AG. Performed the experiments: BP. Analyzed the data: BP, LL, HA, NB, and SC. Contributed reagents/materials/analysis tools: HA.

## Conflict of Interest Statement

The authors declare that the research was conducted in the absence of any commercial or financial relationships that could be construed as a potential conflict of interest.
