# Supplementary material for: Fermented Aloreña Table Olives as a Source of Potential Probiotic Lactobacillus pentosus Strains
Source: Front Microbiol. 2016 Oct 7;7:1583. doi: 10.3389/fmicb.2016.01583 (PMC5054007; doi:10.3389/fmicb.2016.01583)
Supplement: Supplementary file 1 [file Table_1.PDF]

Table S1. Survivability of *Lactobacillus pentosus* strains under different temperature conditions.

Strains	Survival at 4ºC (Log_{10} CFU/ml ± SD*) at different time intervals (days)				Survival at 10ºC (Log_{10} CFU/ml ± SD*) at different time intervals (days)				Survival at 30ºC (Log_{10} CFU/ml ± SD*) at different time intervals (days)			
	**0**	**1**	**3**	**6**	**0**	**1**	**3**	**6**	**0**	**1**	**3**	**6**
Lb. pentosus AP2-11	7,05±0,14^{defghi}	8,95±0,05^{opq}	9,11±0,05^{ghij}	8,86±0,08^{hi}	6,36±0,05^{a}	6,87±0,00^{d}	7,46±0,09^{gh}	7,99±0,03^{fgh}	6,57±0,05^{bc}	8,43±0,01^{d}	8,40±0,00^{m}	7,96±0,06^{hij}
Lb. pentosus AP2-15N	6,00±0,00^{a}	8,55±0,05^{ijk}	8,74±0,31^{bcdefg}	8,53±0,05^{def}	6,45±0,03^{abc}	7,06±0,07^{ghi}	7,35±0,02^{fg}	7,81±0,10^{bcd}	6,90±0,02^{ghij}	8,72±0,08^{hijkl}	8,13±0,06^{ij}	7,80±0,06^{def}
Lb. pentosus AP2-16N	6,95±0,24^{defg}	8,68±0,08^{klm}	8,98±0,10^{efghij}	8,63±0,16^{efg}	6,47±0,05^{abc}	6,73±0,02^{c}	7,14±0,02^{bc}	7,79±0,06^{bc}	6,76±0,12^{de}	8,68±0,10^{ghijkl}	8,06±0,00^{hi}	7,69±0,12^{cd}
Lb. pentosus AP2-17	7,43±0,02^{klmn}	8,96±0,04^{opq}	9,21±0,05^{ij}	9,10±0,01^{k}	6,52±0,08^{bc}	6,94±0,02^{def}	7,00±0,03^{a}	8,09±0,04^{hij}	7,23±0,02^{p}	8,87±0,04^{mn}	8,50±0,12^{n}	8,47±0,08^{n}
Lb. pentosus AP2-18	7,54±0,19^{n}	9,13±0,01^{q}	9,28±0,04^{j}	9,10±0,05^{jk}	7,63±0,04^{n}	7,69±0,09^{pq}	7,73±0,03^{k}	8,06±0,11^{ghij}	7,40±0,04^{q}	8,66±0,01^{ghijk}	8,17±0,03^{jk}	8,26±0,01^{m}
Lb. pentosus CF1-6	7,44±0,11^{klmn}	8,46±0,12^{ghi}	9,08±0,08^{ghij}	8,64±0,12^{efg}	6,58±0,05^{c}	7,05±0,08^{fgh}	7,11±0,03^{abc}	7,87±0,08^{cde}	6,49±0,08^{ab}	8,54±0,04^{defg}	7,96±0,01^{efg}	7,99±0,10^{hij}
Lb. pentosus CF1-20N	6,85±0,21^{cde}	8,07±0,10^{c}	8,61±0,01^{bcde}	8,16±0,06^{b}	6,47±0,01^{abc}	6,47±0,03^{a}	7,58±0,03^{ij}	8,09±0,04^{hij}	6,88±0,05^{fghi}	8,21±0,05^{c}	8,32±0,03^{lm}	8,21±0,01^{lm}
Lb. pentosus CF1-23N	7,02±0,25^{defghi}	8,48±0,03^{hij}	9,08±0,01^{ghij}	8,34±0,12^{c}	6,37±0,16^{a}	7,26±0,01^{klm}	7,69±0,04^{jk}	7,82±0,02^{bcd}	6,98±0,05^{ijkl}	8,18±0,07^{c}	8,15±0,01^{ij}	8,23±0,02^{lm}
Lb. pentosus CF1-30	7,00±0,06^{defghi}	8,66±0,07^{jkl}	9,02±0,05^{fghij}	8,50±0,01^{de}	6,72±0,06^{d}	7,01±0,15^{efg}	7,66±0,10^{jk}	8,05±0,04^{ghij}	6,85±0,07^{efgh}	7,85±0,10^{b}	8,17±0,02^{jk}	8,13±0,03^{kl}
Lb. pentosus CF1-33N	6,92±0,11^{cdef}	8,54±0,09^{hijk}	8,78±0,05^{bcdefg}	8,44±0,08^{cd}	6,46±0,02^{abc}	6,69±0,02^{bc}	7,27±0,02^{def}	7,99±0,07^{fgh}	6,44±0,03^{a}	8,42±0,02^{d}	8,12±0,04^{ij}	7,91±0,03^{fgh}
Lb. pentosus CF1-37N	7,11±0,05^{efghij}	8,71±0,09^{klm}	9,16±0,01^{hij}	8,98±0,08^{ijk}	6,81±0,00^{de}	6,91±0,01^{de}	7,18±0,02^{bc}	7,83±0,01^{cd}	6,67±0,05^{cd}	8,16±0,02^{c}	7,98±0,05^{fgh}	7,79±0,02^{def}
Lb. pentosus CF1-38	6,90±0,08^{cdef}	8,35±0,16^{defgh}	8,69±0,08^{bcdef}	8,68±0,10^{fg}	6,43±0,05^{ab}	6,46±0,04^{a}	7,59±0,05^{ij}	7,71±0,07^{ab}	7,60±0,05^{r}	8,77±0,03^{klmn}	7,88±0,09^{de}	7,83±0,04^{efg}
Lb. pentosus CF1-39	6,89±0,16^{cdef}	8,54±0,07^{hijk}	9,02±0,02^{fghij}	8,45±0,05^{cd}	6,42±0,16^{ab}	6,91±0,02^{de}	7,09±0,03^{ab}	8,00±0,15^{fgh}	7,01±0,01^{jklm}	8,59±0,02^{efgh}	8,11±0,03^{ij}	8,00±0,02^{hijk}
Lb. pentosus CF1-43N	7,29±0,08^{ijklmn}	8,37±0,09^{efghi}	8,82±0,01^{bcdefgh}	8,54±0,01^{def}	6,45±0,02^{ab}	6,60±0,04^{b}	7,09±0,13^{ab}	7,64±0,01^{a}	6,92±0,02^{hijk}	7,59±0,02^{a}	7,14±0,02^{a}	7,00±0,01^{a}
Lb. pentosus CF2-5	6,78±0,00^{cd}	8,87±0,02^{mnop}	8,89±0,02^{cdefghi}	8,57±0,02^{def}	6,92±0,01^{ef}	7,27±0,01^{lm}	7,49±0,02^{hi}	7,81±0,09^{bcd}	6,99±0,04^{ijkl}	8,50±0,07^{def}	7,98±0,04^{fgh}	7,59±0,12^{c}
Lb. pentosus CF2-9	7,15±0,11^{efghijkl}	8,56±0,12^{ijk}	9,01±0,13^{fghij}	8,85±0,12^{hi}	7,23±0,01^{k}	7,16±0,05^{ijk}	7,91±0,07^{lm}	8,07±0,05^{hij}	6,74±0,09^{de}	8,73±0,06^{hijkl}	7,86±0,01^{d}	7,76±0,06^{de}
Lb. pentosus CF2-10N	7,47±0,07^{mn}	8,79±0,02^{lmno}	9,10±0,03^{ghij}	9,04±0,02^{jk}	7,39±0,00^{l}	7,50±0,08^{no}	8,08±0,02^{no}	8,14±0,02^{jk}	7,54±0,02^{r}	8,59±0,02^{efgh}	7,73±0,01^{c}	7,61±0,02^{c}
Lb. pentosus CF2-11	7,24±0,02^{ghijklmn}	8,72±0,05^{klmn}	9,01±0,06^{fghij}	9,07±0,07^{jk}	7,26±0,03^{k}	7,29±0,01^{m}	8,18±0,04^{o}	8,23±0,01^{k}	7,14±0,04^{op}	8,91±0,03^{n}	8,12±0,03^{ij}	8,07±0,06^{jk}
Lb. pentosus CF2-12	6,78±0,25^{cd}	8,45±0,01^{fghi}	8,58±0,07^{bcd}	8,56±0,08^{def}	7,77±0,03^{o}	7,83±0,04^{r}	7,87±0,01^{l}	8,02±0,04^{ghi}	6,80±0,06^{efg}	8,17±0,01^{c}	7,91±0,07^{defg}	7,90±0,02^{fgh}
Lb. pentosus CF2-15G	7,45±0,03^{lmn}	8,87±0,06^{mnop}	8,90±0,04^{cdefghi}	8,65±0,07^{fg}	7,14±0,02^{ijk}	7,20±0,01^{jklm}	7,90±0,05^{lm}	8,08±0,03^{hij}	7,13±0,03^{mnop}	8,81±0,02^{lmn}	8,18±0,01^{jk}	8,04±0,06^{ijk}
Lb. pentosus CF2-15P	7,20±0,12^{fghijklm}	8,69±0,09^{klm}	8,95±0,02^{defghij}	8,74±0,02^{gh}	7,50±0,01^{lm}	7,59±0,07^{op}	7,74±0,04^{k}	8,00±0,01^{fgh}	7,02±0,07^{klmn}	8,73±0,03^{ijklm}	8,26±0,01^{kl}	8,08±0,01^{jk}
Lb. pentosus CF2-20G	6,89±0,27^{cdef}	8,27±0,02^{cdefg}	8,48±0,07^{b}	8,97±0,04^{ijk}	7,09±0,06^{hij}	7,20±0,00^{jklm}	7,46±0,06^{gh}	8,39±0,00^{l}	7,21±0,05^{p}	8,71±0,01^{hijkl}	7,92±0,05^{defg}	7,89±0,07^{fgh}
Lb. pentosus CF2-20P	7,37±0,09^{jklmn}	8,73±0,13^{klmn}	8,77±0,08^{bcdefg}	9,07±0,02^{jk}	7,14±0,06^{ijk}	7,17±0,03^{ijkl}	7,19±0,02^{bcd}	8,42±0,03^{l}	7,16±0,05^{op}	8,67±0,05^{ghijkl}	8,00±0,02^{gh}	7,99±0,03^{hij}
Lb. pentosus LP1N	7,14±0,09^{efghijk}	7,87±0,12^{b}	8,54±0,05^{bc}	8,66±0,10^{fg}	7,05±0,05^{ghi}	7,03±0,09^{fgh}	7,59±0,03^{ij}	8,43±0,00^{l}	7,02±0,14^{klmn}	8,67±0,10^{ghijkl}	7,97±0,07^{efgh}	7,96±0,02^{hij}
Lb. pentosus LP5N	7,25±0,10^{ghijklmn}	8,91±0,03^{nop}	8,77±0,05^{bcdefg}	8,76±0,02^{gh}	6,99±0,03^{fgh}	7,10±0,00^{ghij}	7,32±0,00^{ef}	7,86±0,03^{cde}	6,79±0,08^{efg}	8,60±0,02^{efghi}	7,93±0,05^{defg}	7,94±0,04^{ghi}
Lb. pentosus LP7N	6,98±0,28^{defgh}	7,69±0,12^{b}	9,21±0,46^{ij}	8,96±0,08^{ij}	6,96±0,03^{fg}	7,00±0,09^{efg}	7,99±0,01^{mn}	8,44±0,01^{l}	7,15±0,00^{op}	8,62±0,19^{fghij}	7,34±0,03^{b}	7,34±0,11^{b}
Lb. pentosus LP8N	7,27±0,18^{hijklmn}	8,18±0,10^{cde}	8,66±0,01^{bcdef}	8,65±0,05^{fg}	7,02±0,05^{fghi}	6,96±0,02^{def}	7,08±0,03^{ab}	8,13±0,01^{ijk}	7,06±0,04^{lmno}	8,46±0,04^{de}	7,90±0,05^{def}	7,91±0,01^{fghi}
Lb. pentosus MP-10	7,31±0,11^{ijklmn}	9,00±0,01^{pq}	8,79±0,06^{bcdefgh}	8,54±0,01^{def}	7,53±0,14^{mn}	7,70±0,04^{q}	8,11±0,13^{o}	8,38±0,04^{l}	7,16±0,03^{op}	8,74±0,08^{jklm}	7,88±0,06^{de}	7,89±0,04^{fgh}
Lb. pentosus 2C5	6,75±0,21^{cd}	8,15±0,11^{cd}	8,68±0,15^{bcdef}	8,33±0,10^{c}	7,19±0,04^{jk}	7,45±0,03^{n}	7,50±0,07^{hi}	7,95±0,04^{efg}	7,14±0,06^{nop}	8,47±0,08^{de}	7,75±0,01^{c}	7,62±0,05^{c}
Lb. pentosus 5C2	6,63±0,21^{bc}	8,26±0,21^{cdef}	8,91±0,15^{cdefghij}	8,58±0,00^{def}	7,02±0,04^{fghi}	7,14±0,03^{hij}	7,22±0,02^{cde}	7,90±0,05^{def}	6,86±0,04^{efgh}	8,14±0,02^{c}	7,34±0,03^{b}	7,35±0,07^{b}
Lb. pentosus 5C3	6,39±0,12^{b}	7,15±0,21^{a}	7,54±0,76^{a}	7,00±0,00^{a}	7,04±0,05^{fghi}	7,04±0,04^{fgh}	7,22±0,12^{cde}	8,00±0,02^{fgh}	6,76±0,04def	7,49±0,15^{a}	7,35±0,07^{b}	7,38±0,14^{b}

Table S1 (Continuation). Survivability of *Lactobacillus pentosus* strains under different temperature conditions.

Strains	Survival at 37ºC (Log_{10} CFU/ml±SD*) at different time intervals (days)				Survival at -80ºC (Log_{10} CFU/ml±SD*) at different time intervals (days)			
	0	1	3	6	0	1	3	6
Lb. pentosus AP2-11	6,57±0,05^{bc}	8,27±0,00^{lm}	7,68±0,02^{ghi}	6,99±0,04^{c}	8,91±0,04^{ghi}	8,80±0,01^{mn}	8,82±0,17^{ijkl}	8,95±0,07^{op}
Lb. pentosus AP2-15N	6,90±0,02^{ghij}	8,14±0,02^{ijk}	7,86±0,07^{lmn}	7,45±0,03^{ef}	8,57±0,02^{cd}	8,56±0,01^{ij}	8,78±0,03^{ijk}	8,96±0,02^{op}
Lb. pentosus AP2-16N	6,76±0,12^{de}	8,18±0,03^{jkl}	7,71±0,04^{hij}	7,47±0,18^{ef}	8,85±0,07^{fgh}	8,72±0,00^{klmn}	8,83±0,01^{ijkl}	8,90±0,01^{no}
Lb. pentosus AP2-17	7,23±0,02^{p}	8,21±0,02^{kl}	7,97±0,03^{no}	7,91±0,05^{m}	8,54±0,05^{c}	8,46±0,03^{gh}	8,52±0,02^{fg}	8,63±0,07^{jk}
Lb. pentosus AP2-18	7,40±0,04^{q}	8,41±0,01^{no}	7,84±0,11^{klm}	7,80±0,16^{jklm}	8,66±0,00^{de}	8,59±0,03^{ij}	9,10±0,02^{no}	8,83±0,04^{mn}
Lb. pentosus CF1-6	6,49±0,08^{ab}	7,50±0,03^{b}	7,62±0,04^{gh}	7,88±0,01^{lm}	8,82±0,01^{fg}	8,77±0,02^{lmn}	8,93±0,04^{l}	8,86±0,08^{mno}
Lb. pentosus CF1-20N	6,88±0,05^{fghi}	8,04±0,04^{ghi}	7,57±0,02^{efg}	7,52±0,03^{efg}	8,84±0,01^{fg}	8,35±0,01^{f}	8,07±0,04^{cd}	9,33±0,00^{r}
Lb. pentosus CF1-23N	6,98±0,05^{ijkl}	7,93±0,09^{f}	7,20±0,00^{b}	7,24±0,02^{d}	8,78±0,01^{f}	8,20±0,07^{e}	8,22±0,08^{e}	9,33±0,02^{r}
Lb. pentosus CF1-30	6,85±0,07^{efgh}	8,03±0,09^{gh}	7,48±0,03^{de}	7,41±0,01^{e}	8,36±0,08^{b}	8,13±0,02^{de}	8,07±0,03^{cd}	9,08±0,00^{q}
Lb. pentosus CF1-33N	6,44±0,03^{a}	7,76±0,07^{cd}	7,82±0,03^{jkl}	7,80±0,01^{jklm}	8,76±0,00^{ef}	8,59±0,00^{ij}	8,55±0,03^{fg}	8,76±0,16^{lm}
Lb. pentosus CF1-37N	6,67±0,05^{cd}	8,10±0,01^{hij}	7,49±0,06^{de}	7,77±0,00^{jkl}	9,18±0,00^{mnopq}	8,69±0,01^{kl}	9,07±0,11^{mn}	9,05±0,10^{pq}
Lb. pentosus CF1-38	7,60±0,05^{r}	8,05±0,01^{ghi}	7,57±0,13^{efg}	7,63±0,04^{ghi}	8,66±0,03^{de}	8,51±0,01^{ghi}	8,77±0,16^{hijk}	8,71±0,05^{kl}
Lb. pentosus CF1-39	7,01±0,01^{jklm}	8,07±0,01^{ghi}	7,82±0,01^{jkl}	7,72±0,04^{ijk}	8,86±0,01^{fgh}	8,44±0,11^{fg}	8,86±0,03^{jkl}	8,77±0,05^{lm}
Lb. pentosus CF1-43N	6,92±0,02^{hijk}	7,33±0,01^{a}	6,73±0,03^{a}	6,48±0,12^{b}	8,98±0,13^{ijk}	8,22±0,04^{e}	8,43±0,01^{f}	8,34±0,02^{g}
Lb. pentosus CF2-5	6,99±0,04^{ijkl}	8,61±0,02^{q}	7,75±0,12^{ijkl}	7,57±0,13^{fgh}	9,08±0,03^{klmn}	8,50±0,02^{ghi}	8,93±0,04^{l}	8,85±0,06^{mno}
Lb. pentosus CF2-9	6,74±0,09^{de}	8,21±0,00^{kl}	7,61±0,13^{fgh}	7,14±0,05^{d}	9,15±0,11^{mnop}	8,43±0,05^{fg}	8,72±0,07^{hi}	8,63±0,01^{jk}
Lb. pentosus CF2-10N	7,54±0,02^{r}	7,98±0,01^{fg}	7,61±0,01^{fgh}	7,17±0,01^{d}	9,19±0,05^{nopq}	8,65±0,04^{jk}	8,94±0,06^{lm}	8,81±0,05^{lmn}
Lb. pentosus CF2-11	7,14±0,04^{op}	8,48±0,09^{op}	7,96±0,03^{mno}	6,89±0,02^{c}	9,07±0,03^{klm}	9,12±0,02^{q}	9,09±0,06^{n}	9,08±0,03^{q}
Lb. pentosus CF2-12	6,80±0,06^{efg}	8,47±0,01^{op}	7,72±0,02^{hijk}	7,79±0,01^{jklm}	8,25±0,04^{ab}	7,99±0,08^{c}	8,04±0,07^{c}	8,00±0,04^{e}
Lb. pentosus CF2-15G	7,13±0,03^{mnop}	8,34±0,03^{mn}	8,05±0,06^{op}	7,69±0,03^{hij}	9,25±0,03^{pq}	9,05±0,06^{q}	9,23±0,03^{o}	8,92±0,12^{no}
Lb. pentosus CF2-15P	7,02±0,07^{klmn}	8,33±0,04^{mn}	8,12±0,06^{p}	7,80±0,00^{jklm}	8,23±0,02^{a}	7,84±0,01^{b}	7,34±0,03^{a}	7,19±0,04^{a}
Lb. pentosus CF2-20G	7,21±0,05^{p}	8,20±0,06^{kl}	7,69±0,02^{ghi}	7,73±0,01^{ijk}	9,24±0,05^{pq}	8,54±0,07^{hi}	8,18±0,04^{de}	8,15±0,04^{f}
Lb. pentosus CF2-20P	7,16±0,05^{op}	7,90±0,07^{ef}	7,77±0,01^{ijkl}	7,85±0,04^{klm}	9,26±0,00^{q}	8,91±0,01^{p}	8,64±0,09^{gh}	8,45±0,01^{gh}
Lb. pentosus LP1N	7,02±0,14^{klmn}	7,93±0,02^{ef}	7,69±0,09^{ghi}	7,90±0,06^{lm}	8,76±0,01^{ef}	8,04±0,03^{cd}	8,03±0,06^{c}	7,72±0,05^{d}
Lb. pentosus LP5N	6,79±0,08^{efg}	8,19±0,01^{jkl}	7,75±0,03^{ijkl}	7,85±0,05^{klm}	9,16±0,02^{mnopq}	7,49±0,02^{a}	7,68±0,08^{b}	7,51±0,00^{b}
Lb. pentosus LP7N	7,15±0,00^{op}	7,71±0,06^{c}	7,38±0,03^{cd}	7,63±0,01^{ghi}	9,02±0,03^{jkl}	8,73±0,07^{klmn}	8,73±0,03^{hij}	8,51±0,01^{hi}
Lb. pentosus LP8N	7,06±0,04^{lmno}	7,83±0,04^{de}	7,50±0,01^{ef}	7,90±0,01^{lm}	8,92±0,04^{ghij}	8,59±0,03^{ij}	8,11±0,01^{cde}	7,70±0,01^{cd}
Lb. pentosus MP-10	7,16±0,03^{op}	8,56±0,01^{pq}	7,61±0,06^{fgh}	7,71±0,10^{ij}	9,21±0,16^{opq}	8,89±0,08^{op}	8,90±0,12^{kl}	8,57±0,03^{ij}
Lb. pentosus 2C5	7,14±0,06^{nop}	8,04±0,01^{gh}	7,34±0,00^{c}	6,28±0,03^{a}	9,20±0,08^{opq}	8,74±0,08^{klmn}	8,52±0,00^{fg}	8,10±0,04^{ef}
Lb. pentosus 5C2	6,86±0,04^{efgh}	8,08±0,13^{ghi}	7,36±0,05^{c}	6,57±0,07^{b}	9,11±0,05^{lmno}	8,81±0,03^{no}	8,71±0,06^{hi}	8,55±0,08^{hij}
Lb. pentosus 5C3	6,76±0,04^{def}	7,91±0,06^{ef}	7,85±0,02^{lmn}	7,50±0,02^{efg}	8,96±0,07^{hij}	8,71±0,03^{klm}	7,59±0,02^{b}	7,60±0,05^{bc}

±SD: standard deviations of three independent experiments.

*: Different subscripts lowercase letters represent significant differences according to 2-sided Tukey´s HSD between strains ($p < 0.05$).

Strains with a slight decrease in viable cell counts after six days storage at -80ºC are marked in blue.
