# Supplementary material for: Fermented Aloreña Table Olives as a Source of Potential Probiotic Lactobacillus pentosus Strains
Source: Front Microbiol. 2016 Oct 7;7:1583. doi: 10.3389/fmicb.2016.01583 (PMC5054007; doi:10.3389/fmicb.2016.01583)
Supplement: Supplementary file 2 [file Table_2.PDF]

Table S2. Technological properties of *Lactobacillus pentosus* strains.

Strains	Degradation of antinutritive compounds		Degradation of different energy sources							Enzymes		
	Raffinose	Stachyose	Glucose	Fructose	Galactose	Saccharose	Lactose	Lactulose	Inulin	HDC	BSH	CA
Lb.pentosus AP2-11	+	-	++	++	+	++	++	++	-	+	+	+
Lb.pentosus AP2-15N	+	-	++	++	+	++	++	++	-	+	+	+
Lb.pentosus AP2-16N	+	-	++	++	+	++	++	++	-	+	+	+
Lb.pentosus AP2-17	+	-	++	++	+	++	++	++	-	+	+	+
Lb.pentosus AP2-18	+	-	++	++	+	++	++	++	-	-	+	-
Lb.pentosus CF1-6	-	-	++	++	+	++	++	++	-	+	+	-
Lb.pentosus CF1-20N	-	-	++	++	+	++	++	+	-	-	+	+
Lb.pentosus CF1-23N	-	-	++	++	+	++	++	+	-	+	+	-
Lb.pentosus CF1-30	-	-	++	++	+	++	++	+	-	-	+	+
Lb.pentosus CF1-33N	-	-	++	++	+	++	++	++	-	+	+	-
Lb.pentosus CF1-37N	-	-	++	++	+	++	++	++	-	+	+	-
Lb.pentosus CF1-38	-	-	++	++	+	++	++	++	-	+	+	-
Lb.pentosus CF1-39	+	-	++	++	+	++	++	++	-	-	+	+
Lb.pentosus CF1-43N	-	-	++	++	+	++	++	++	-	+	+	+
Lb.pentosus CF2-5	+	-	++	++	+	++	++	++	-	+	+	-
Lb.pentosus CF2-9	+	-	++	++	+	++	++	++	-	+	+	+
Lb.pentosus CF2-10N	+	-	++	++	+	++	++	++	-	-	+	-
Lb.pentosus CF2- 11	+	-	++	++	+	++	++	++	-	+	+	-
Lb.pentosus CF2-12	-	-	++	++	-	++	-	-	-	-	+	+
Lb.pentosus CF2-15G	+	-	++	++	+	++	++	++	-	+	+	-
Lb.pentosus CF2-15P	+	-	++	++	+	++	++	++	-	+	+	-
Lb.pentosus CF2-20G	-	-	++	++	+	++	++	++	-	-	+	+
Lb.pentosus CF2-20P	+	-	++	++	+	++	++	++	-	-	+	-
Lb.pentosus Lp-1N	-	-	++	++	+	++	++	++	-	+	+	-
Lb.pentosus Lp-5N	-	-	++	++	+	++	++	+	-	-	+	-
Lb.pentosus Lp-7N	-	-	++	++	+	++	-	++	-	-	+	-
Lb.pentosus Lp-8N	-	-	++	++	+	++	++	++	-	+	+	-
Lb.pentosus MP-10	+	+	++	++	+	++	++	++	-	+	+	-
Lb.pentosus 2C5	-	-	++	++	+	++	++	++	-	-	+	-
Lb.pentosus 5C2	+	-	++	++	+	++	++	++	-	-	+	+
Lb.pentosus 5C3	+	-	++	+	+	++	-	++	-	-	+	-

HDC, haeme-dependent catalase; BSH, bile salt hydrolase; CA, cellulolytic activity.
